# Composite genotypes of progestogen-associated endometrial protein gene and their association with composition and quality of dairy cattle milk

**DOI:** 10.5713/ab.20.0596

**Published:** 2021-02-15

**Authors:** Magdalena Kolenda, Beata Sitkowska, Dariusz Kamola, Barry D. Lambert

**Affiliations:** 1 Department of Animal Biotechnology and Genetics, Faculty of Animal Breeding and Biology, UTP University of Science and Technology, Bydgoszcz 85-084, Poland; 2 Department of Physiological Sciences, Faculty of Veterinary Medicine, Warsaw University of Life Sciences, Warsaw 02-776, Poland; 3 Department of Animal Science and Veterinary Technology, Tarleton State University, Stephenville, TX 76402, USA

**Keywords:** β-Lactoglobulin, Cattle, Genetics, Progestogen-associated Endometrial Protein (PAEP), Polymorphisms, Milk Performance

## Abstract

**Objective:**

The progestogen-associated endometrial protein (*PAEP*) gene encodes the main whey protein in milk, β-lactoglobulin. The aim of the study was to investigate polymorphism in the *PAEP* gene and its association with milk yield, composition, and quality.

**Methods:**

Test-day records for 782 dairy cows were analysed. A total of 10 single nucleotide polymorphisms (SNP) within the *PAEP* gene were investigated. The following parameters were recorded: milk yield (MY, kg/d), percent milk fat (%), protein (PP, %), dry matter (DMP, %) and lactose (LP, %), urea content (UC, mg/L) as well as natural logarithm for somatic cell count (LnSCC, ln). Effect on genomic estimated breeding values accuracy was evaluated with pedigree and single step model.

**Results:**

Results show that only three SNPs were polymorphic, creating 5 composite genotypes: P1 to P5. Differences in MY between composite genotypes were noted in the two tested herds. Cows with P5 composite genotypes were characterised by the highest PP and LnSCC and the lowest LP and UC (p<0.05). P4 was linked to an increased DMP and UC, while P3 to an increase in LP and decrease in PP and LnSCC. Both factors are important markers in herd management and have high influences on the herds economics. For 5 out of 7 traits the accuracy of prediction was improved by including the haplotype as a fixed effect.

**Conclusion:**

Presented results may suggest a new way to optimise breeding programmes and demonstrate the impact of using genomic data during that process.

## INTRODUCTION

The dairy market is rapidly developing and implementing many modern technologies [1]. One of worldwide trends in cattle breeding is genomic assessment. Cow genome analysis and comparison to milk parameters provides the opportunity to precisely analyse associations between various haplotypes or composite genotypes and performance traits. The value of genomic analysis is widely recognised by the dairy industry and, as a result, many research papers have been published on association of different genotypes with milk and milking performance traits [2–4]. It has been shown that mutations in the sequence of various milk protein genes may cause changes in protein expression, milk yield (MY) and milk composition. Occurrence of alleles that were linked with favourable results in the population may be altered by selecting sires through DNA analysis [2]. The search for quantitative trait loci (QTL) and single nucleotide polymorphism (SNP) located in candidate genes may lead to identification of genetic markers associated with important traits that may be used in marker-assisted selection [5]. Such selection is often used to increase the frequency of preferable alleles in the population in order to gain economic benefits. Producers may also incorporate genomic selection into their breeding programmes in order to reduce generation intervals and improve the overall performance of their herds [6].

There are over 50,208 QTLs associated with milk production traits in the CattleQTLdb database, out of which 19,782 are linked to milk protein content [7]. Milk proteins are known to have a role in nutritional properties of milk. Among the best-known milk protein genes are: 4 casein genes (*CSN1S1*, *CSN2*, *CS1S2*, and *CSN3*) and β-lactoglobulin gene, also called progestogen-associated endometrial protein gene (*PAEP*) [8]. Moreover, *PAEP* has been indicated as a candidate gene that is associated with MY as well as quality of milk [9]. CattleQTLdb lists a total of 14 traits and 53 QTLs associated with *PAEP* gene, among which 12 QTLs are associated with milk beta-lactoglobulin content and percentage [7]. PAEP is localised on the 11th bovine chromosome, is composed of 6 coding exons, and encodes the main whey protein [10]. β-Lactoglobulin is found in milk of most mammalian species. It also accounts for approximately 50% of whey protein and 10% of the total milk protein in bovine milk [8,11]. Throughout the years many genetic variants of the gene have been identified [12,13].

The present study uses the data obtained during a routine breeding value estimation. Microarray data are used for genomic estimated breeding values (GEBV) evaluation, however, authors decided to investigate the additional relationship between PAEP SNPs and phenotypes. A selection of different PAEP SNPs is added to microarrays, however, not all of them may have a significant impact on milk performance; therefore, it is important to investigate whether these SNPs, also as composite genotypes, significantly affect milk performance. If PAEP SNPs have a large effect on traits of interest their inclusion as fixed effect in the model could improve accuracy of GEBVs and they could also be used in breeding programmes relying on pedigree and genotyping just a few SNPs to support breeding decisions.

The aim of the study was to investigate polymorphisms in the *PAEP* gene with the use of microarray genotyping data gathered during the process of routine estimation of breeding value as well as its association with yield, composition and quality of milk of Polish Black and White Holstein-Friesian cows.

## MATERIALS AND METHODS

In accordance with Resolution No. 13/2016 of the National Ethics Committee for Animal Experiments (Poland) of June 17, 2016, the consent of the Ethics Committee is not required for the collection of animal material for genotyping.

### Animals

The study was carried out on three Polish Black and White Holstein-Friesian dairy herds (A–C). Herds were considered to be among the best in Poland in terms of MY. A total of 782 dairy cows were used in the study. Cows were fed a total mixed ration and had access to feed *ad libitum*.

### Data collection

Milk recording data were collected in the period from 2016 to 2019 according to A4 method (the interval between two successive recordings of two daily milkings ranging between 28 and 33 days) accredited by the International Committee for Animal Recording (ICAR) [14], by the Milk Analysis Laboratory of the Polish Federation of Cattle Breeders and Dairy Farmers (PFCB&DF) which is certified by ISO 17025 and certified by ICAR. All data were stored and provided by SYMLEK IT system (by PFCB&DF). Genetic data used in this study were collected in the process of routine estimation of breeding value (EBV) that is performed in Poland. Poland as a member of the Euro Genomics cooperative uses customised EuroGenomics arrays. In the present study the following versions were used: Eurogenomics v3_POL; Eurogenomics v4_POL; Eurogenomics v5_POL; Eurogenomics v6_POL; Eurogenomics v8b_POL; Eurogenomics MD_POL with Infinium HD Illumina protocol. The different panels had very little overlap and due to limited number of genotypes available the imputation was not successful, thus only 6,765 genome wide SNPs with call rate >0.95 across all animals and minor allele frequency >0.001 were used.

**Figure 1:**
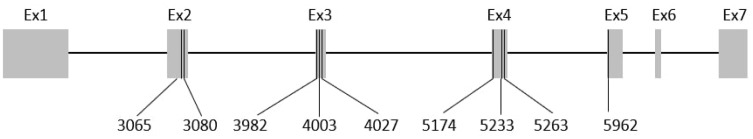
Nine progestogen-associated endometrial protein (*PAEP*) gene single nucleotide polymorphisms described in the present study with their nucleotide location within PAEP. Nucleotide position on the gene is presented according to *Bos taurus* reference genome ARS-UCD1.2. Ex1–Ex7 represents the number of PAEP exon.

Biopsy samples (ear punch) were collected in the period from 2015 to 2019. Eurogenomics arrays’ probe list and sequences are confidential and could not be published in the present paper. For the purpose of the present study authors have chosen SNPs that were present on all array versions. Farmers have authorised the authors to access a portion of the SNP data. Genotyping data provided by PFCB&DF and farmers were evaluated and 10 polymorphisms within PAEP (in exons 2, 3, 4, and 5) were identified (Figure 1, Table 1).

### Statistical analysis

Allele and genotype frequencies for all SNPs were calculated and data was subjected to the Hardy-Weinberg equilibrium test. The national milk recording scheme (SYMLEK system) provided test day records for somatic cell counts (SCC, ths/mL) in the milk of all studied cows. SCC data were log transformed to the natural logarithm for somatic cell count (LnSCC).

A dataset containing 9,284 records on milk performance was analysed. The following parameters were recorded during the research: MY (kg/d), percentage of fat (FP, %), protein (PP, %), dry matter (DMP, %) and lactose (LP, %), urea content (UC, mg/L) as well as natural LnSCC (ln). Moreover, a dataset containing information on PAEP genotypes was added into the analysis.

In order to check the validity of the use of haplotypes for GEBV estimation, two models were used: with and without haplotypes in pedigree based and single-step calculations. The 7-trait model (repeatability) was used with fixed effects of herd, year-season of the sampling, year-season of calving, sample number (related to days in milk), sample number squared and age of cow at sampling; with the last three effects fitted as covariates. For this purpose, free Fortran 90 software was used [15]. A total of 191 cows born in 2017 was selected for the validation group. The method of partial vs whole data was used based on the research carried out by Legarra and Reverter [16] who showed that the correlation of EBVs in validation animals between the whole and partial data set reflects ratio of the accuracies of the two models. The model was run with pedigree as the relationship matrix and as a single step model. Analysed traits were statistically characterised. The impact of respective PAEP composite genotypes on tested parameters was assessed by one-way analysis of variance followed by the Bonferroni post-test. p-Values less than 0.05 were considered as statistically significant (SAS software package) [17].

## RESULTS AND DISCUSSION

### Allele and genotype frequencies

In the present study 10 genetic variants (SNPs) were investigated and allele and genotype frequencies were calculated (Table 2). It was noted that seven genotypes were monomorphic, while three (PAEP_3982, PAEP_5174, PAEP_5263) showed genetic diversity. CT genotype was the most frequent amongst PAEP_3982 genotypes (50%), with CC and TT being less frequent (30% and 20%, respectively). In our study, Hardy-Weinberg equilibrium test was performed showing observed and expected heterozygosities at the same levels (0.30), chi^2^ at the level of 0.208. p Value (0.649) was greater than α = 0.05, which indicates that, regarding PAEP_3982 SNP, the studied population was consistent with Hardy-Weinberg equilibrium. Similarly, 49% of all tested cows were heterozygous in terms of PAEP_5174, while 32% had TT and 19% CC genotypes. Population was in Hardy-Weinberg equilibrium as chi2 (0.0145) and p value (0.904) did not allow this hypothesis to be disregarded. Results showed that 49% of all individuals were heterozygous for PAEP_5263, the Hardy-Weinberg equilibrium test revealed that the population was also consistent with Hardy-Weinberg equilibrium (p = 0.904). In the present study, seven out of ten SNPs were monomorphic, which may suggest that due to extensive breeding and selection, genetic diversity for these SNPs was reduced. Other authors also have investigated PAEP variants, however most focused mainly on variants A and B, which are characterised by a number of different SNPs [6,12,18], among others three SNPs that were detected to be polymorphic in the present study. Comparing the data showing nucleotide presence in each variant, it can be noted that variant A is characterised by the presence of thymine in PAEP_3982, cytosine in PAEP_5174 and thymine in PAEP_5263, while variant B has cytosine in the position 3,982, thymine in 5,174 and cytosine in 5,263. Hristov et al [12] reported that the frequencies of A and B alleles differ according to cattle breed: for Shorthorn Rhodopean cattle breed AA and BB frequencies were low (0.063) and heterozygote frequency was very high (0.88), while in Bulgarian Rhodopean cattle the frequency of AA genotype was higher (0.40) and AB lower (0.58). Also, Barbosa et al [9] investigated polymorphisms within the *PAEP* gene revealing two alleles and three genotypes. They reported that within the population of Girolando cows, most common were heterozygotes (0.48) and noted that genotype frequencies were in Hardy-Weinberg equilibrium (chi^2^ = 1.04; p<0.01).

Moreover, out of the three PAEP variants (PAEP_3982, PAEP_5174, PAEP_5263) that showed polymorphism, 5 composite genotypes were formed (Table 3): P1 (PAEP_3982CC +PAEP_5174TT+PAEP_5263CC, giving the composite genotype CCTTCC), P2 (CTCTCT), P3 (CTTTCC), P4 (TTCCTT), P5 (TTCTCT). The most frequent composite genotype in the studied population was P2, with 48% animals having that composite genotype, while P3 and P5 were least frequent (these composite genotypes were found only in 2% of the tested population). Further studies on a larger population may further help with analysing the frequencies of created composite genotypes.

### The effect of PAEP genotypes on tested traits

The relative increase in correlation calculated based on partial vs whole data by including haplotypes in the GEBV calculation is presented in Table 4. In 5 out of 7 analysed traits, some improvement in accuracy of the EBVs by incorporating haplotypes was found with an average of 15.8% for pedigree based EBVs and 9.5% for single step GEBVs increase in accuracy across the traits. Overall, the accuracy was low because the population structure was not favourable and the genetic relationship between training and validation was very low, also the amount of available data used in the training was small. With the relative gains for MY, UC, and SCC in the range of 30% for pedigree-based analysis, genotyping three SNPs necessary to determine haplotypes may be beneficial for breeding programmes with limited resources that can not afford the use of microarrays. However, validation on a larger dataset and in the target population is recommended.

Significant differences (p<0.05) were found between different PAEP composite genotypes regarding tested traits. Differences between composite genotype groups (P1 to P5) in relation to different parameters are presented in Table 5. Animals with P5 composite genotype were characterised by the highest (p<0.05) values for PP and LnSCC and the lowest for LP and UC (p<0.05). P4 composite genotype was linked with increased DMP and UC, while P3 was associated with increased LP as well as decreased PP and LnSCC. While no statistical differences were noted while studying tested herds as one group, differences in MY between composite genotypes were noted in Herd A and B. These results are in accordance with our previous reports; for instance, Sitkowska et al [19] who investigated polymorphism in the *PAEP* gene reported that PAEP genotypes have an impact on MY and milk composition. They noted that animals with BB genotype were characterised by a higher FP and PP in milk, while allele A was associated with MY. Similarly, Dokso et al [13] noticed that MY of animals with AA genotype was higher by 9.58% than for BB genotype, while cows homozygous for B allele had a higher FP (by 4.28%) than AA animals. Wageh Zaglool et al [4] linked AA genotype to a higher MY and PP (by 16.81% compared to BB animals) and BB with higher FP in milk (by 30.95% compared to AA animals). These authors did not report significant differences in LP between different genotypes.

Monitoring UC in milk of dairy cattle may help farmers to detect health issues that may result in reduced fertility of their cows. Concentration of milk urea provides information on the efficiency of protein synthesis, as well as on the balance between crude protein and energy. As urea dissolves in milk, milk urea concentration is strongly correlated with blood urea nitrogen level. A high level of UC suggests an inefficient protein conversion. On the other hand, low UC may cause health and fertility problems [20]. Literature suggests that the optimal UC in cow’s milk is between 150 and 300 mg/L [20,21]. In the present study UC did not exceed 300 mg/L (on the average UC was 245.01 to 270.1 mg/L) (Table 5). Lower values were reported by Czajkowska et al [21] (206.4 mg/L) and Rzewuska and Strabel [20] (230.1 mg/L).

The SCC is an important parameter that has a great economic impact since higher levels of somatic cells are associated with mastitis [22]. In the present study, a significant association between PAEP composite genotypes and LnSCC was recorded in the whole population as well as in herd A and C, indicating that animals with P5 composite genotype were characterised by the highest and P3 by the lowest LnSCC (Table 5). In herd B, the same tendency was observed, however, the differences were not statistically significant.

It is worth noticing that selected herds for this study were among the best in Poland and where farmers have incorporated genomic selection into their breeding programmes for many years. Their work may contribute to the fact that MY obtained by tested cows was higher than many mean values for the average dairy cattle herd. For instance, while authors of the present study reported MY to be 37.69 to 38.40 kg/d, the average MY in 2018 in Poland was 27.21 kg/d [23]. Mulder et al [24] noted MY to be even lower: 24.31 kg/d. Soyudal et al [6], who investigated the influence of respective PAEP alleles on milk performance of Holstein Friesian cows in Turkey, reported MY in 305-day lactation to be 28.90 kg/d for animals with genotype AA, 27.86 kg/d kg for heterozygotes and 26.36 kg/d for BB. These levels were considerably lower than those reported in the present study. The differences between MYs in different herds may result from many factors, including different management. Herds selected in the present study are considered as one of the best in Poland in terms of MY, and the owners have put an emphasis on a careful breeding management, that was also supported by genotyping data.

Microarray genotyping in dairy herds is used mostly for EBV, the data on specific SNPs is often not further investigated. Confirming the impact of selected PAEP SNPs that are included in the microarrays and their haplotypes may allow farmers to obtain information on genotypes related to only these SNPs that are relevant for their production systems. It is worth mentioning that many farmers decide not to genotype their herds with microarrays due to high costs. EBV performed based on pedigrees combined with genetic markers evaluation is more accurate than EBV that does not include information on genetic markers. Therefore confirming the impact of PAEP SNPs and their haplotypes on milk performance may allow farmers to obtain relevant genetic information in more cost-efficient way as genotyping three SNPs would be cheaper than genotyping with the use of microarray. Fitting the haplotypes as fixed effects in addition to the presence of PAEP SNPs in the construction of the H matrix also provided some gains in accuracy which suggests that the effect of these haplotypes is larger than assumed by a single step GBLUP model.

## CONCLUSION

Based on the results of the present study it can be noted that polymorphism analysis is needed to monitor the level of SNP variability within herds. In the study, 10 different SNPs in the *PAEP* gene were analysed. Three of them were shown to be polymorphic and importantly related to production traits. Presented data show a positive correlation between P4 composite genotype in herd A and MY as well as a positive correlation between P3 with lower LnSCC level. Both of these factors are very important markers in herd management and have high influences on overall herd economics. Presented results show the impact of using genomic data beyond the relationship matrix for the benefit of breeding programmes.

## Figures and Tables

**Table 1:** Progestogen-associated endometrial protein information (ARS-UCD1.2)

SNP name	Position on *PAEP* gene	Position on the chromosome	SNP	AA change	Exon
PAEP_3065	3,065	103257028	G>C	Glu/Gly	2
PAEP_3080	3,080	103257043	C>T	Pro/Ser	2
PAEP_3982	3,982	103257948	C>T	n/a	3
PAEP_4003_1	4,003	103257969	G>C	Lys/Asn	3
PAEP_4003_2	4,003	103257969	G>T	Lys/Asn	3
PAEP_4027	4,027	103257993	C>G	Ile/Met	3
PAEP_5174	5,174	103259143	C>T	n/a	4
PAEP_5233	5,233	103259202	A>G	Glu/Gly	4
PAEP_5263	5,263	103259232	C>T	Ala/Val	4
PAEP_5962	5,962	103259931	C>T	Pro/Leu	5

PAEP, progestogen-associated endometrial protein; SNP, single nucleotide polymorphisms; A, adenine; C, cytosine; G, guanine; T, thymine; Ala, alanine; Glu, glutamic acid; Gly, glycine; Ile, isoleucine; Lys, lysine; Leu, leucine; Met, methionine; Asn, asparagine; Pro, proline; Ser, serine; Val, valine; n/a, synonymous.

**Table 2:** Allele and genotype frequency (%) in *PAEP* gene

SNP name	Allele’s nucleotide		Genotype frequency			Allele frequency	
		
	A	B	AA	AB	BB	A	B
PAEP_3982	C	T	0.30	0.50	0.20	0.55	0.45
PAEP_5174	C	T	0.19	0.49	0.32	0.43	0.57
PAEP_5263	C	T	0.32	0.49	0.19	0.57	0.43
PAEP_3065	G	C	1.00	0.00	0.00	1.00	0.00
PAEP_3080	C	T	0.00	0.00	1.00	0.00	1.00
PAEP_4003_1	G	C	1.00	0.00	0.00	1.00	0.00
PAEP_4003_2	G	T	0.00	0.00	1.00	0.00	1.00
PAEP_4027	C	G	1.00	0.00	0.00	1.00	0.00
PAEP_5233	A	G	1.00	0.00	0.00	1.00	0.00
PAEP_5962	C	T	0.00	0.00	1.00	0.00	1.00

*PAEP*, progestogen-associated endometrial protein; SNP, single nucleotide polymorphisms.

**Table 3:** Progestogen-associated endometrial protein composite genotype frequencies (%)

Composite genotype	Nucleotide composition				PAEP composite genotype frequency

	PAEP_3982	PAEP_5174	PAEP_5263	Haplotype	
P1	CC	TT	CC	CCTTCC	0.29
P2	CT	CT	CT	CTCTCT	0.48
P3	CT	TT	CC	CTTTCC	0.02
P4	TT	CC	TT	TTCCTT	0.19
P5	TT	CT	CT	TTCTCT	0.02

PAEP, progestogen-associated endometrial protein.

**Table 4:** Correlation between composite genotypes and phenotypes

Milk parameters	Pedigree	Single-step
Milk yield (kg/d)	27.9	17.1
Fat content (%)	−7.3	−6.9
Protein content (%)	5.8	5.8
Dry matter content (%)	−4.6	−6.2
Lactose content (%)	*	*
Urea content (mg/L)	30	21
LnSCC (ln)	43	26.4

LnSCC, logarithm for somatic cell count.

**Table 5:** Impact of progestogen-associated endometrial protein composite genotypes on milk parameters (p<0.05)

Composite genotypes	Milk yield (kg/d)	Fat content (%)	Protein content (%)	Dry matter content (%)	Lactose content (%)	Urea content (mg/L)	LnSCC (ln)
Whole population							
Average	38.30	3.75	3.36	12.80	4.86	254.73	4.23
P1	38.40	3.72	3.33^ad^	12.72^ab^	4.86	248.49^b^	4.15^ad^
P2	38.30	3.75	3.36^c^	12.80^b^	4.87^cd^	251.19^a^	4.16^be^
P3	37.69	3.70	3.28^be^	12.71	4.89^ab^	261.21	4.05^cf^
P4	38.22	3.79	3.40^cde^	12.88^a^	4.84^ac^	270.10^abc^	4.44^defg^
P5	38.13	3.83	3.42^ab^	12.87	4.82^bd^	245.01^c^	4.51^abcg^
Herd A							
Average	37.24	3.98	3.43	13.19	4.92	288.17	4.48
P1	37.22^a^	3.92^a^	3.37^bd^	13.04^be^	4.92	286.66	4.45^a^
P2	36.80^bc^	4.06^ab^	3.47^de^	13.32^def^	4.94^ab^	283.57^a^	4.31^be^
P3	33.74^abd^	3.93	3.34^ce^	13.01^cf^	4.92	284.96	4.20^cf^
P4	38.28^cd^	3.90^bc^	3.43^a^	13.08^ad^	4.90^a^	295.16^a^	4.61^def^
P5	35.29	4.35^c^	3.64^abc^	13.69^abc^	4.84^b^	282.60	5.44^abcd^
Herd B							
Average	41.14	3.54	3.24	12.49	4.88	232.20	3.90
P1	40.71^a^	3.55	3.23	12.46	4.86^a^	230.70	3.90
P2	41.03^b^	3.57	3.25	12.52	4.88^a^	232.93	3.89
P3	42.72	3.39	3.18	12.31	4.89	231.06	3.85
P4	42.43^ab^	3.49	3.26	12.46	4.86	235.46	3.96
P5	40.99	3.47	3.28	12.39	4.86	215.44	3.99
Herd C							
Average	34.04	3.96	3.51	13.09	4.80	272.87	4.65
P1	33.95	4.01	3.53	12.18	4.83^ab^	272.25	4.58
P2	33.44	3.94	3.51	13.06	4.79^a^	269.02^a^	4.65
P3	34.82	3.92	3.36	12.94	4.87	278.30	4.15^a^
P4	34.84	3.94	3.50	13.05	4.80^b^	278.63^a^	4.71
P5	35.23	4.14	3.54	13.26	4.77	273.49	4.94^a^

LnSCC, logarithm for somatic cell count.

a–g Values in columns marked with the same letters are statistically different (p<0.05).
